# Is it ethical to prescribe generic immunosuppressive drugs to renal transplant patients?

**DOI:** 10.1186/s40697-014-0023-8

**Published:** 2014-09-09

**Authors:** Julie Allard, Marie-Chantal Fortin

**Affiliations:** Centre de recherche du Centre hospitalier de l′Université de Montréal (CRCHUM), 900 Saint-Denis Street, Montreal, Quebec H2X 0A9 Canada; Nephrology and Transplantation Division, Centre hospitalier de l’Université de Montréal (CHUM), 1560 Sherbrooke Street East, Montreal, Quebec H2L 4 M1 Canada; Department of Social and Preventive Medicine, Bioethics Program, School of Public Health, Université de Montréal, Montreal, Canada

**Keywords:** Immunosuppressive drugs (ISDs), Ethics, Transplantation, Generic drugs

## Abstract

**Purpose of the review:**

This review was conducted to determine the ethical acceptability of prescribing generic immunosuppressive drugs to renal transplant patients.

**Sources of information:**

The literature search was conducted using Pubmed and Google Scholar.

**Findings:**

The use of generic immunosuppressive drugs (ISDs) in transplantation is a controversial topic. There is a consensus among transplant societies that clinical data is lacking and that caution should be exercised. The reluctance to use generic ISDs in organ transplantation is partly related to the fact that most are “critical dose drugs”, and that either low dosing or overdosing could have serious adverse consequences for both patients and society (i.e., the loss of scarce organs). In this paper, we examine the various ethical issues involved such as distributive justice, physician duties, risks versus benefits, conflict of interest, informed consent, and logistical and economic issues.

**Limitations:**

Our analysis was limited by the paucity of clinical data on generic ISDs and the absence of health economics studies to quantify the benefits of prescribing generic ISDs.

**Implications:**

Our study led us to conclude that it would be ethical to prescribe generic ISDs provided certain conditions were met. These include regulatory safeguards to minimize the risks of substitution; education of patients; and further clinical and health economics studies to better inform clinicians, patients and society of the risks and costs related to drug substitution.

## Abrégé

**But de l’article:**

Le but de cet article est de questionner s’il est éthique de prescrire des immunosuppresseurs génériques aux patients transplantés d’un rein.

**Sources d’informations:**

Nous avons effectué une recension des écrits en utilisant Pubmed et Google Scholar.

**Résultats:**

La prescription d’immunosuppresseurs génériques en transplantation rénale est un sujet controversé. Les sociétés savantes sont en accord sur le fait qu’il y a un manque de données cliniques et que la prudence est de mise. Une des explications à cette attitude réfractaire est le fait que les immunosuppresseurs sont considérés comme des « médicaments à dose critique ». Par conséquent, leur surdosage ou leur sous-dosage peut avoir des conséquences désastreuses pour le patient et la société (i.e., la perte d’un greffon). Dans cet article, nous examinerons les enjeux éthiques impliqués dans ce débat tels que la justice distributive, les devoirs des médecins, l′analyse des risques et des bénéfices, le conflit d’intérêt, le consentement éclairé ainsi que des enjeux logistiques et économiques.

**Limites:**

Les données cliniques existantes sont insuffisantes et limitent notre analyse. De plus, l′absence d’analyse économique sur l′utilisation d’immunosuppresseurs génériques constitue une autre limite à notre étude.

**Implications:**

Suite à cette analyse, nous pouvons conclure qu’il serait éthique de prescrire des immunosuppresseurs génériques dans certaines circonstances. Celles-ci incluent la mise en place de mesures qui minimisent les risques de substitution, l′éducation des patients et la réalisation d’études cliniques et économiques afin de mieux informer les cliniciens, les patients et la société sur les risques reliés à la prescription de ces médicaments ainsi qu’aux coûts associés.

## What was known before?

The prescription of generic ISDs is controversial. There is a concern that the bioequivalence acceptability range could lead to generic drift.

## What this adds

This review enhances existing knowledge with an analysis of the ethical issues related to the prescription of generic ISDs.

## Why is this review important?

This review explores the ethical issues related to the prescription of generic ISDs. This is particularly topical and important given the recent presence of generic ISDs on the market.

## What are the key messages?

Generic ISDs are economical but could pose a theoretical risk to transplant patients. If risks are reduced by safeguard policies and if their use is shown to be clearly economic and safe, prescribing generic ISDs would be ethical.

## Implications for future research/policy

Our review leads us to conclude that a comprehensive cost-effectiveness analysis of generic ISDs and clinical studies in *de novo* and stable transplant patient for every new generic ISD are necessary.

## Introduction

Renal transplantation is one of the greatest medical advances of the twentieth century, improving chances of survival and quality of life for end-stage renal disease patients. This medical success has been made possible through immunosuppressive drugs (ISDs) that prevent graft rejection. Patients are required to take these drugs for the graft life span. Although ISDs decrease graft rejection, they are associated with multiple adverse effects. They are also expensive: the annual cost of immunosuppressive treatment can be as high as C$15,000 per patient [1]. According to the Canadian Institute for Health Information, the wholesale value of Canadian purchases of ISDs grew more quickly between 2004 and 2010 than purchases in any other major therapeutic category [2]. The recent arrival on the Canadian market of generic ISDs could lower these costs. The Institut national d’excellence en santé et en services sociaux (INESS), a provincial organization in Quebec with the mission to promote clinical excellence and the efficient use of resources in the health and social services sector, recently published a Notice to the Minister, refusing to exempt the brand-name versions of tacrolimus from Quebec’s lowest price policy [3]. However, the use of generic ISDs raises many ethical questions regarding distributive justice, physician responsibilities, conflict of interest and informed consent. In this paper, we look more closely at these issues, as well as various position statements on generic ISDs. We do not examine situations related to specific immunosuppressive drugs.

## Review

### Generic immunosuppressive drugs

Although generic drugs are widely accepted in the medical community, the situation with ISDs is different, as many of them are “critical dose drugs”. Health Canada defines critical dose drugs as “those drugs where comparatively small differences in dose or concentration lead to dose- and concentration-dependent, serious therapeutic failures and/or serious adverse drug reactions” [4]. Health Canada recognizes only nine critical dose drugs, three of which are commonly used ISDs: cyclosporine, tacrolimus and sirolimus [4]. These drugs are monitored closely by blood concentration dosage to achieve an optimal therapeutic effect.

Since generic cyclosporine was introduced in the US market in 1995, the majority of articles on the use of generic ISDs have highlighted the controversial nature of this issue. A 2005 article by Taber et al., reporting a significantly higher incidence of acute rejection in patients who received generic cyclosporine versus those who received the brand-name drug, contributed to the generic controversy [5]. Even though the authors reported significant limitations to their research, including a higher intra-patient variation for the generic drug group, this study has probably influenced the way transplant professionals view generics.

A generic drug has the same active ingredient as a brand-name drug, but the excipients can be different, which could affect the absorption of the active ingredient and lead to a different blood concentration. To be approved by Health Canada, a generic of a critical dose drug has to meet strict criteria of pharmaceutical equivalence and bioequivalence with the innovator product (90% to 112% of the area under the curve). Since blood concentration is so important for critical dose drugs, generic ISDs and innovator products are not considered freely substitutable, even when they have been demonstrated to be bioequivalent. There is also a concern about the bioequivalence of different generic drugs: every generic has to be proven to be bioequivalent to the innovator product, but not to other generic products. There is a theoretical possibility of generic “drift”, i.e., a generic at one end of the acceptable range of the area under the curve (AUC) might not be bioequivalent to another generic at the other end of the acceptable range [6]. The main concern about the arrival of generic drugs on the market is the possibility of uncontrolled substitutions (i.e., substitutions that are made without informing the prescriber), which could lead to under- or over-immunosuppression caused by generic drift, both of which could lead to devastating complications. As new generics enter the market, patients could even face successive substitutions, thus increasing fluctuations in drug concentrations—a known factor in poor long-term outcomes following kidney transplantation [7].

Certain bioequivalence studies on the kidney transplant population support the safe use of generic ISDs [8–11]. However, there are also reports of observed variations in drug concentration following drug substitution [5,12]. Both sets of studies are small without long-term follow-up. While the evidence is not sufficiently robust to serve as a basis for medical decisions, these studies have probably had a significant impact on physicians’ confidence in generic ISDs. They have bolstered the arguments of detractors and have likely contributed to a widespread reluctance to prescribe generic ISDs.

Many authors nonetheless believe that the reluctance to use generics is based on misconceptions regarding the approval process [13]. Even though there is a theoretical possibility of generic drift, a review of 2,070 bioequivalence studies of all drugs approved from 1996 to 2007 by the FDA showed that, in reality, the average difference was comparable to the variability between two batches of the innovator drug [14]. Some authors believe that eventually all generic drugs will be demonstrated to be freely substitutable, following ongoing research based on new bioequivalence study designs [6,15].

### Position statements on generic immunosuppressive drugs

The Canadian Society of Transplantation (CST) issued recommendations in April 2012 regarding the use of generic ISDs, following a thorough review of the available scientific literature. The CST recommended extreme caution and estimated that the use of generic ISDs posed a significant patient safety risk, because of the lack of safety evidence and the absence of structural safeguards to prevent uncontrolled substitutions. They asked for bioequivalence to be demonstrated, not only in healthy adults, but also in transplant recipients and in subpopulations known to have a high variability in blood concentration [1].

The American Society of Transplantation (AST) held a conference in 2001 to review the data and issues regarding the use of the generic ISDs, and published a summary of the meeting in 2003 [16]. Participants in the forum strongly supported efforts to offer less expensive medications, hoping to improve compliance. Most agreed that the prescription of generic ISDs *de novo* is safe in low-risk transplant recipients. Some expressed concerns about uncontrolled substitutions, and there was strong support for bioequivalence studies in at-risk subpopulations. It is worth mentioning that in the US context, ISDs are reimbursed only during the first three years post-transplant. Cessation of treatment because of financial strain is a known cause of graft failure [17].

The European Society of Organ Transplantation (ESOT) commissioned an Advisory Committee to formulate recommendations on the use of generic drugs in solid organ transplant recipients [18]. The ESOT was satisfied with the stricter criteria issued by the European Medicines Agency (EMA), which match those of Health Canada. The EMA is not opposed to the use of generic drugs that meet the new criteria, but the agency proposes to regulate generic substitutions of critical dose drugs in vulnerable patient populations.

The Société francophone de transplantation also issued recommendations after generic ISDs were introduced in the market without consulting the medical transplant community. They supplemented the ESOT recommendations, calling for stricter criteria for bioequivalence, prescription of generic ISDs by their commercial name, and the monitoring of patients taking generic ISDs in order to collect clinical data [19]. Table 1 summarizes the position statements of these different transplant societies.

**Table 1:** **Position statements of various professional societies regarding the use of generic immunosuppressive drugs (ISDs)**

**Canadian Society of Transplantation (2012)** **[** 1 **]**	• Insufficient literature regarding efficacy and safety.
	• Close monitoring with any change.
	• Not recommended in pediatric patients.
	• The intended drug formulation must be explicitly stated on all prescriptions to avoid substitutions.
	• Educate patients about formulations and substitutions.
	• Prescriber and patient should be involved in any decision to change formulation. Mandatory notification of the prescriber should be a legal requirement.
	• Licensing requirements for critical dose drugs must be re-assessed. Bioequivalence in solid organ transplant recipients (SOTR). Requirement for generic manufacturers to provide clinical outcome data in SOTR.
	• Transplant centres should be funded according to the increased costs associated with managing SOTR arising from the introduction of generic immunosuppression.
**American Society of Transplantation (2003)** **[** 16 **]**	• Supports the availability of efficacious, less expensive immunosuppressive medications and endorses efforts to introduce generic alternatives. Medication costs may contribute to non-compliance with prescribed medical regimens.
	• FDA-approved generic immunosuppressive agents appear to provide adequate immunosuppression to low-risk patients.
	• Insufficient data to make recommendations for at-risk populations (African-Americans or pediatric).
**European Society for Organ Transplantation (2011)** **[** 18 **]**	• Generic formulations that do not meet the stricter criteria should not be used.
	• Substitution should only be initiated by the transplant physician; pharmacists or insurance providers should refrain from forcing substitution.
	• Repetitive substitution should be avoided.
	• Patients should be informed about substitution and taught how to identify different formulations of the same drug so they can alert their physician if an uncontrolled substitution is made.
	• The simultaneous use of different formulations in the same patients should be avoided.

### Ethical issues

In this section, we examine in further detail the various ethical issues related to the use of generic ISDs in renal transplantation.

#### Distributive justice

Theories of distributive justice aim to describe how a society or group should allocate its scarce resources or products among individuals with competing needs or claims [20]. Since money is a scarce resource in every healthcare system, and individuals with different illnesses have competing needs, what is the best response? There are several theories proposing principles to help decide on how to allocate resources. These include utilitarian, egalitarian, libertarian and communitarian theories, and the more recent capabilities and well-being theories [21]. However, none of these theories has resulted in a consensus around principles [21,22]. It is beyond the scope of this article to review all of these theories. Since Canada’s health system is based on egalitarian values, one could attempt to use egalitarian theory to resolve the allocation problem. The egalitarian approach would involve equal opportunities for all patients to obtain effective treatment [22]. It would also mean reinvesting the resources saved by the use of generic ISDs to help the most disadvantaged patients in transplantation. Egalitarian principles would be respected if generic ISDs produced good clinical outcomes, allowing other patients an opportunity to be treated.

#### Physician duties

The situation with critical dose drugs is complex. Even though Health Canada acknowledges the bioequivalence of generic drugs, many physicians still believe there is some risk involved. Previous reports of increased incidence of acute rejection with generic cyclosporine partly explain this stance [5]. Physicians who are reluctant to prescribe generic ISDs are bound by their duty of beneficence towards patients and are also the stewards of a scarce resource (organs). For many, no degree of risk is acceptable.

Physicians’ dual responsibilities (to their patients and society) could be conflicting [23]. As Pellegrino writes, the Hippocratic Oath binds physicians collectively “to duties to other physicians and to individual patients. Neither the Oath nor the other books of the Hippocratic Corpus mention social obligations of broader kinds, such as responsibility for the availability, accessibility, and affordability of health care, or collective responsibility for public health, the poor, or public policy” [24]. While some authors call for a redefinition of the physician’s role that would include a duty toward society, others believe the two are irreconcilable [25,26]. In a study by Beach and colleagues, 70% of physicians felt that their main responsibility was toward individual patients rather than society [27]. Also, in a review of quantitative surveys on physicians’ attitudes toward rationing, it has been shown that the latter are generally willing to consider cost in clinical decisions and are in favour of cost containment; however, when asked about cases in which the aims and consequences of cost containment are specified, their support decreases noticeably [28]. These findings are not surprising, given that physicians have a personal relationship with their patients who want to feel that their well-being comes first. Physicians are conscious of the need for cost containment, but would rather not see benefits withheld from their patients. Maynard states that in a public healthcare system, prioritization is in fact determined by physicians who provide the treatments they consider most appropriate, not necessarily in relation to need or comparative cost-effectiveness, as should be the case [29].

#### Risk-benefit analysis (RBA)

Risk-benefit analyses of the use of generic ISDs vary from country to country. In the United States, for instance, it has been documented that patients who have to pay for brand-name ISDs bear economic burdens that truly affect their lives; the out-of-pocket expenses for these ISDs lead to non-compliance, graft loss and adverse complications [17]. Seeing their patients’ distress, physicians might be more likely to prescribe generic ISDs. For Canadian patients, the economic burdens of brand-name medication are far less onerous. Prescribing generics would likely not significantly increase compliance or decrease adverse events resulting from non-compliance. Even if some individuals were relieved by reduced treatment costs, the greatest advantages would be to other patients and to the healthcare system, which would benefit from additional available resources. These benefits are, however, extremely difficult to quantify. It is also very difficult to ascertain the risks, given the few available studies in the literature [8-12,30-33]. There is a small theoretical risk of a variation in the drug concentration. Even though this risk is unquantifiable, the consequences could be disastrous for both patients and society. Under-immunosuppression could lead to graft loss while over-immunosuppression could lead to neoplastic complications or life-threatening infections. From an individual perspective, an RBA analysis may not support the use of generic ISDs in Canada, since individuals derive no obvious benefits and may incur some risk. From a wider societal perspective, however, an RBA analysis might show benefits to be obtained through potential cost savings.

In the UK, the National Health Service is seeking ways to reinvest efficiency savings into clinical services in order to improve the quality of patient care [34]. These strategies have led some transplant centres to use generic tacrolimus and generic mycophenolate mofetil in both *de novo* and stable renal transplanted patients [34].

While this paper is focused on renal transplantation, the same questions have been raised in thoracic transplantation as well [35]. The risk acceptability may be lower in thoracic transplantation, as there is no alternative treatment like dialysis. Using the precautionary principle, substitution could be started in renal transplantation and extended to thoracic and other life-saving organ transplantation if post-approval clinical data of the generic ISDs showed solid clinical equivalence.

#### Conflict of interest

The use of generic drugs also raises the issue of conflict of interest. Pharmaceutical companies are omnipresent in the medical community, and manufacturers of brand-name ISDs are also very active in the transplantation world. These companies offer grants and speaker honoraria; they are trusted, as they have been in the business for a while and have invested in long-term relationships with physicians. They also sponsor professional organizations in the field of transplantation. It has been documented that even though 61% of physicians believe their decisions are not influenced by drug companies’ marketing strategies, only 16% have the same confidence in their colleagues [36]. A review of the literature on conflict of interest and drug companies showed that physician-industry interactions do affect professional behaviour and prescribing [37]. The review also showed that these interactions reduce the prescription of generic drugs. It is also worth mentioning that some scientific papers that promote the use of generic ISDs are sponsored by generic drug companies [8]. It is important to take these conflicts of interest into account in the debate around the use of generic ISDs.

#### Patients’ informed consent

Many campaigns aim to promote the use of generic drugs or to reassure the population when substitution is mandatory, as is the case under the public prescription drug insurance plans of many provinces and most private insurance plans in Canada [38]. The market share of generic drugs in this country has been increasing over the last decade, reaching 63% in 2012 [39], partly because of changes in government and insurance programs, but also because of public confidence. When prescribing a generic ISD, physicians must inform their patients that the drug in question may be different from other generic drugs. Efforts are needed to educate patients about the possible effects of substitution. Patients must also be made aware of the appearance of their medication in order to detect and report any substitution. This education is necessary to obtain patients’ informed consent. If the information is not presented in a nuanced manner, it could potentially undermine much-needed public trust in the generic approval process.

Should well-informed patients refuse to take generic ISDs, their autonomy would be respected. However, under most insurance plans, they would have to cover the cost difference between the generic and brand-name drug. Some might argue that the opportunity for patients to exercise their autonomy depends on their income, as many would not be able to afford the brand-name drug. But this could be argued for any generic drug as well, and if we were to rank autonomy higher than societal health by covering the cost of preferred versions of the drug, there would be a greater loss in health coverage, since generic drugs save billions of dollars annually. Such a decision would also run counter to values enshrined in the *Canada Health Act* such as universality and accessibility. The mandatory substitution policy is in accordance with Daniels’ theory of fair equality of opportunity [22] in that it provides an opportunity for all to access treatment while allowing them the liberty to purchase a brand-name drug.

#### Logistical and economic issues

Unless it is eventually demonstrated that every generic ISD is freely substitutable, there is a need for regulations that would minimize the risks of uncontrolled substitutions. In Canada, as in most developed countries, pharmacists may automatically substitute a medication without necessarily notifying the physician [35]. Physicians may write “no substitution” on the prescription; however, depending on the insurance plan, the patient could be responsible for the cost difference between the two brands. Physicians, pharmacists and regulators would have to work together to set new rules around generic substitutions, and then educate their respective professional communities.

While generic ISDs are invariably cheaper than brand-name drugs, their use is not necessarily economical. Since all professional associations recommend close monitoring of any substitutions [1,16,18,40], the costs of increased monitoring for both medical staff and patients (time, lost wages, travel to hospital) must be evaluated. It is worth noting that increased monitoring would probably only be necessary in the short term, and costs would be lowered once the substitution was proven to be safe and effective. Substitution could therefore prove to be an economical choice in the long term. There is also a consensus around the need for increased patient education, which comes at a cost. The CST has openly asked for funds to be allocated to transplant centres to meet these new needs [1]. As stated earlier, there would be costs associated with implementing new regulations and providing information to physicians and pharmacists. A comprehensive cost-benefit analysis including these new costs is needed to assess the cost-effectiveness of generic ISDs.

The use of generic ISDs may also involve logistical issues around drug shortages. In Canada, most provinces have policies to regulate generic prices. Although these price cap policies may be economical in the short term, they may also have been a factor in recent drug shortages [41]. As the prices are lowered, it becomes less profitable to manufacture the drugs and a shortage of generic ISDs could be disastrous.

## Conclusion

To determine whether it is ethical to prescribe generic ISDs, we recommend a comprehensive cost-effectiveness analysis to ascertain the benefits, as well as clinical studies in *de novo* and stable renal transplant patients for every generic ISD. We recommend minimizing the risks that could be associated with generic drift by implementing new policies such as a law prohibiting non-physicians (pharmacists, insurance providers, etc.) from authorizing substitutions of brand-name ISDs with generic ISDs, or substitutions between generic ISDs. If generic ISDs are demonstrated to be clinically safe and more cost-effective than brand-name drugs, and if policies are implemented to minimize the risks, it would be ethical to use them, since the benefits to society would be greater than the risks to individual patients.
